# Nonastreda multimodal dataset for efficient tool wear state monitoring

**DOI:** 10.1016/j.dib.2025.111905

**Published:** 2025-07-23

**Authors:** Hubert Truchan, Zahra Ahmadi

**Affiliations:** a L3S Research Center, Leibniz University Hannover, Appelstr. 4, 30167 Hannover, Niedersachsen, Germany; b Peter L. Reichertz Institute for Medical Informatics, Hannover Medical School, Karl-Wiechert-Allee 3, 30625 Hannover, Niedersachsen, Germany

**Keywords:** Time series, Images, Classification, Regression, Remaining useful life, Anomaly detection

## Abstract

With advancements in artificial intelligence (AI), there is a growing need to bridge the gap between multimodal learning capabilities and the availability of high-quality datasets for tool wear estimation. Industrial scenarios frequently require domain-specific knowledge, specialized datasets, and efficient deployment on resource-constrained edge devices that demand minimal memory, low latency, and optimized computational performance. While there has been a shift from unimodal sensor-based approaches to multisensory, multimodal strategies, this transition remains in its early stages. Developing feature extraction methods, multimodal fusion techniques, and correlation analysis frameworks is crucial for improving tool wear prediction models.

Existing multimodal open-source datasets have several limitations in addressing these challenges:

• They are often restricted to a specific set of data modalities, limiting adaptability.
• They primarily feature general-purpose objects, which are not well-suited for industrial applications requiring specialized domain knowledge.
• They lack support for lightweight models designed for real-time processing on edge devices.
• They lack in-depth documentation or dedicated data loaders, limiting reproducibility.

To bridge this gap, we introduce the **Nonastreda Multimodal Dataset** for efficient tool wear state monitoring. The dataset models the multimodal nature of tool wear progression in industrial milling processes, integrating nine data modalities. It comprises 512 samples, each containing RGB images of the shaft milling tool, workpiece, and material chip, along with three scalograms and three spectrograms derived from force signals. Data collection was performed using ten milling tools in an industrial production environment.

The dataset is designed to support classification tasks (sharp, used, dulled) and regression tasks predicting three target variables: flank wear (µm), gaps (µm), and overhang (µm). Each sample can be analyzed independently or as part of a temporally correlated sequence.

Accompanying scripts for data processing and analysis are available in the repository.

Specifications Table

**Table:** 

Subject	Computer Sciences
Specific subject area	Industrial flank tool wear of the milling machine
Type of data	Image: .jpg tool, chip, spectrogram; .png scalograms and workpiece Raw: .mat forces_xyz_raw
Data collection	The Nonastreda dataset is obtained from a real industrial milling device processing material with a shaft milling tool. During the milling process, time-series and image data were collected to model industrial tool wear. • Images were captured using an industrial unit microscope. • Three force signal sequences (Fx, Fy, Fz) were collected using: industrial dynamometer, amplifier, bus coupler, industrial PC. • Workpiece Material: C45 Tempered Steel. • Tool Type: End Mill.
Data source location	*L3S Research Center, Leibniz University Hannover,* *Appelstr. 4, 30,167 Hannover, Niedersachsen, Germany*
Data accessibility	Repository name: Mendeley Data Data identification number: 10.17632/m892d2wtzh.1 Direct URL to data: https://data.mendeley.com/datasets/m892d2wtzh/1 *Data overview:* *Converting forces_xyz_raw.mat into spectrograms, scalograms or wavelets:*https://github.com/hubtru/Girape/tree/main/scripts *Scripts compiling the data:* https://github.com/hubtru/Girape/tree/main https://github.com/hubtru/Impala/tree/main
Related research article	*none*

## 1 Value of the Data

• Nonastreda is the first multimodal dataset with nine data modalities modeling a real milling process. It includes RGB images of the milling tool, material chips, and workpiece, along with time-series force signals in x, y, and z axes. This dataset allows for reproduction of experiments, extraction of novel features, and the development of advanced machine learning models for tool wear monitoring and predictive maintenance.
• The Nonastreda dataset enables both classification (sharp, used, dulled) and regression tasks (predicting flank wear [µm], gaps [µm], and overhang [µm]), supporting both unimodal and multimodal approaches. Additionally, it facilitates various subtasks, including:
  ○ multilabel regression,
  ○ remaining useful life (RUL) estimation,
  ○ anomaly detection,
  ○ analysis of sampling rate, noise and drift (particularly for the Fz component),
  ○ zero-shot tool wear classification,
  ○ and feature engineering.
• The dataset includes raw time-series force signals in x, y, and z axes, along with precomputed scalograms and spectrograms, enabling researchers to explore novel time-frequency signal representations. These force signals allow for multimodal analysis and serve as an indirect tool wear estimation method, capturing variations in cutting dynamics and providing insights into tool-material interactions. By leveraging the Girape/scripts github repository, researchers can generate custom spectrograms, scalograms, wavelets, and other signal-processing transformations.
• Researchers can extract a broad range of new features using time-series analysis techniques, including:
  ○ Fourier Transforms for frequency-domain representation.
  ○ Discrete and Continuous Wavelet Transforms (e.g., Morlet, Shannon, Gaussian) for multi-resolution analysis.
  ○ Signal decomposition techniques, such as Empirical Mode Decomposition (EMD) or Variational Mode Decomposition (VMD).
  ○ Domain-specific features, e.g., cutting force variability and spectral content analysis.
  ○ Harmonic analysis to examine periodic force signal components.
  ○ Spectral Kurtosis and Envelope Analysis for tool wear and damage detection.
  ○ Cross-correlation features to study interactions between force signals and visual observations.
• The dataset provides a foundation for future research directions, such as:
  ○ Developing an automated diagnostic feature selection framework for identifying the most informative features for tool wear classification.
  ○ Extracting and analyzing blade shapes from RGB tool images, enabling direct estimation of flank wear. These images allow the development of new segmentation techniques, cutting edge line analysis, and advanced image-processing methods for precise wear measurement.
  ○ Enhancing tool wear classification and regression models by leveraging single modalities for optimizing downstream tasks (e.g., using force signals for early anomaly detection or RGB images for precise wear segmentation) and exploring multimodal approaches to develop more effective fusion techniques for integrating diverse sensor data.
  ○ Incorporating raw force signals (Fx, Fy, Fz) into multimodal analysis, expanding insights into cutting dynamics.
  ○ Deriving additional modalities from raw force signals for enhanced modeling of wear progression.
  ○ Exploring anomaly detection, RUL estimation, and signal drift measurement in industrial milling processes.
  ○ Performing correlation analysis across different modalities, improving the interpretability of multimodal fusion models.
  ○ Exploring the transferability of multimodal approaches to other industrial settings and different types of machining processes.
  ○ Applying instance-level normalization of signals and images to improve robustness against data distribution shifts.
  ○ Developing tailored augmentation techniques to increase data variability.

## 2 Background

The condition of a milling tool plays a critical role in ensuring high-quality production, particularly in the manufacturing of precision-engineered components. Tool wear directly affects dimensional accuracy, surface roughness, and production efficiency, leading to deviations from design specifications. Manufacturers must balance replacement costs with the risk of defective parts, making tool wear monitoring essential for cost-effective production.

Several open-source datasets have contributed to tool wear research, each offering different sensor modalities and machining conditions. The newly released dataset from cuboidal milling operations supplies synchronized vibration and spindle-current signals, enabling tool-fault classification and prediction [1]. The QIT-CEMC 2025 dataset provides multimodal data, including force, torque, sound, and vibration, for coated end milling cutters [2]. The Mudestreda dataset focuses on force spectrograms and tool images for single-flank wear estimation [3], while the MATWI dataset incorporates force, vibration, and acoustic emission data for carbide tools [4]. Other datasets, such as NJUST-CCTD, provide image-only data for tool wear classification [5], whereas SDU-QIT [6] and IEEE NUAA_IDEAHOUSE [7] include vibration and power signals for wear estimation. Additionally, datasets like the Milling Data Set [8] and PHM2010 [9] focus on multimodal wear prediction with force, vibration, and acoustic emission signals.

Despite their contributions, many of these datasets lack detailed documentation, preprocessing scripts, or multimodal fusion support, limiting their usability [10]. The Nonastreda dataset addresses these gaps by integrating nine sensor modalities, including RGB images and force signals, for both direct and indirect tool wear estimation. With classification and regression labels, along with ready-to-use preprocessing scripts, Nonastreda facilitates unimodal and multimodal research, supporting the development of AI-driven tool wear monitoring systems.

## 3 Data Description

**Fig. 1:**
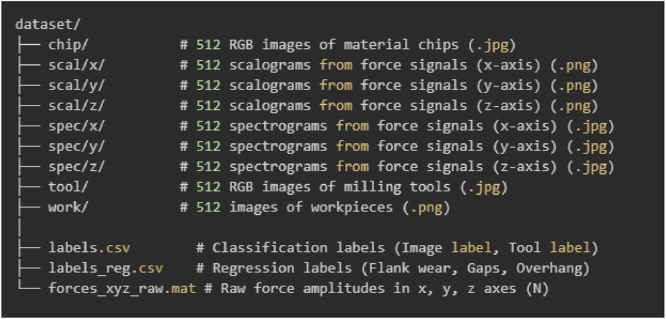
Nonastreda dataset structure and file organization.

The Nonastreda dataset consists of nine modalities representing the milling tool wear process, from the shaft milling tools, structured into multiple subfolders and data files. The dataset includes images, time-frequency representations (scalograms, spectrograms), raw force signals, and structured labels for classification and regression tasks. Fig. 1 presents a detailed breakdown of the dataset components.

### 3.1 Image data

The dataset contains three sets of RGB images capturing different aspects of the milling process:

• Chip images (chip/): 512 RGB images representing material chips generated during milling.
• Tool images (tool/): 512 RGB images of the milling tool, capturing visible wear.
• Workpiece images (work/): 512 PNG images showing the final machined workpiece surface.

### 3.2 Time-frequency representations

The dataset includes scalograms and spectrograms, which are precomputed time-frequency transformations of raw force signals along three axes:

• Scalograms (scal/x/, scal/y/, scal/z/): 512 PNG images per axis, obtained from wavelet transforms of force signals.
• Spectrograms (spec/x/, spec/y/, spec/z/): 512 JPEG images per axis, derived from short-time Fourier transforms (STFT).

### 3.3 Labels and ground truth

**Fig. 2:**
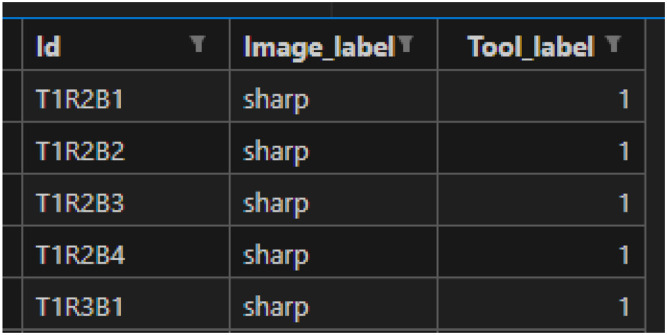
Labels.csv: classification labels.

Classification Labels (labels.csv) contains 512 entries, where each row includes an instance ID, an Image label (sharp, used, dulled), and a Tool label (1 for tool ID). The preview of the first five rows is presented in Fig. 2.

Regression Labels (labels_reg.csv) contains 512 numerical labels, mapping each instance ID to three continuous wear measurements:

• Flank wear (µm)
• Gaps (µm)
• Overhang (µm)

**Fig. 3:**
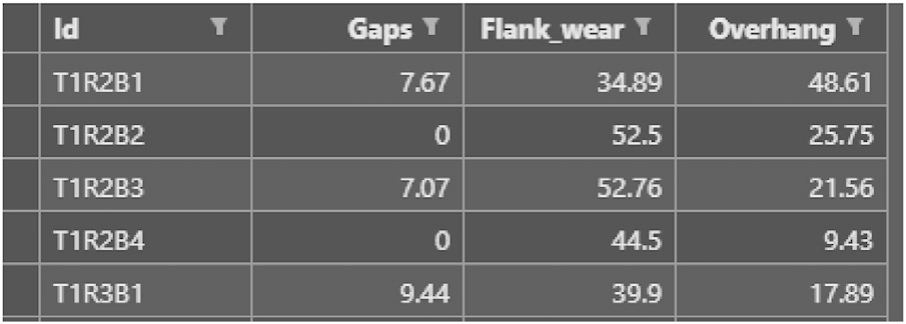
Labels_reg.csv: regression labels.

The preview of the first five rows is presented in Fig. 3.

### 3.4 Raw force signals

**Fig. 4:**
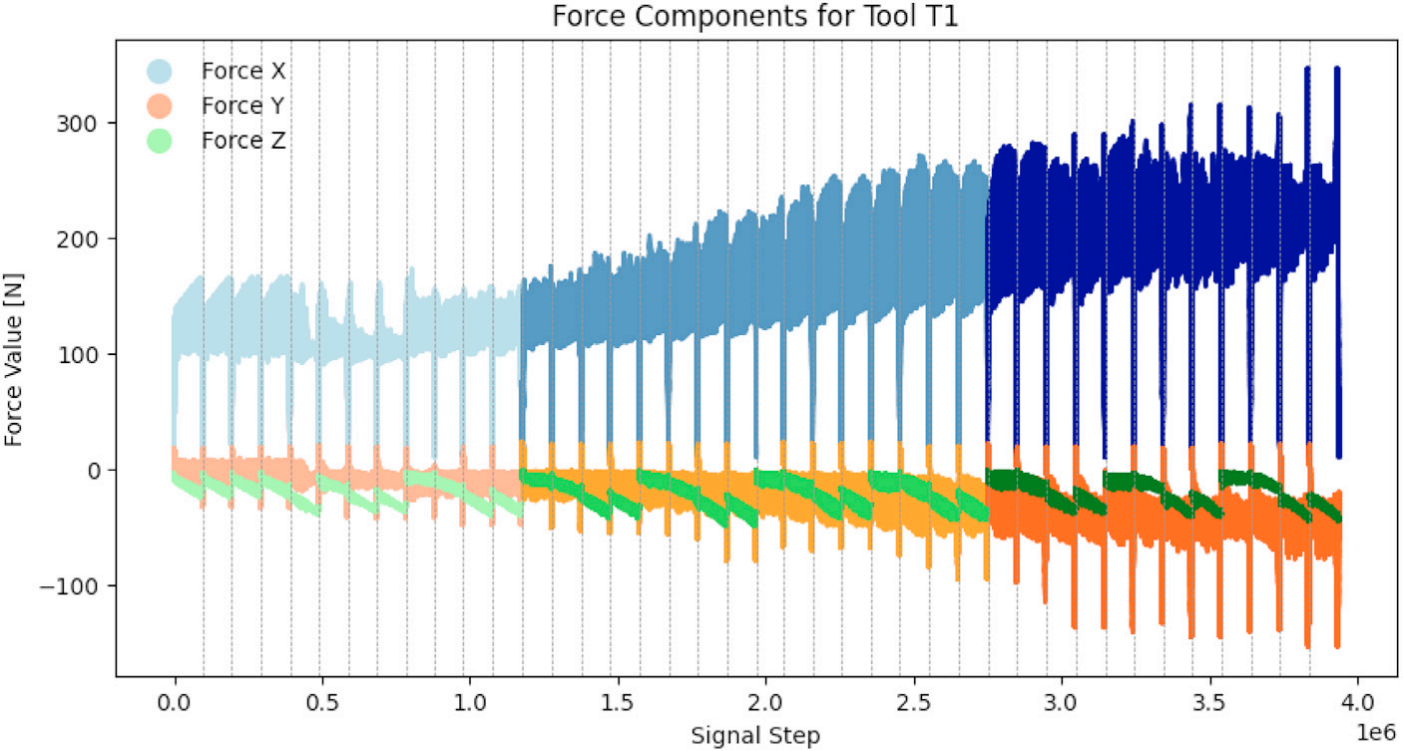
forces_xyz_raw.mat: raw force signals of tool T1. Colour changes indicate interclass transitions: sharp, used and dulled. Dashed horizontal lines indicate transitions between instances.

The forces_xyz_raw.mat file contains raw force amplitudes (N) in the x, y, and z axes, providing an unprocessed at a sampling frequency of 1 kHz with no missing data. Visualizations of the forces of tool T1 is presented in Fig. 4.

### 3.5 Validation strategy

**Fig. 5:**
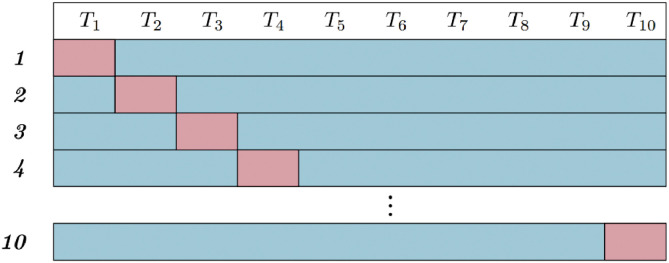
The 10 fold cross-validation setup. Red are marked test tools and blue are marked train and validation tools.

To ensure robust model evaluation, we recommend using a 10-fold cross-validation setup, as illustrated in Fig. 5. The dataset consists of data collected from 10 different milling tools (T1-T10). In each fold, instances from nine tools are used for training and validation, while the remaining tool is reserved for testing. This process is repeated ten times, with each tool serving as the test set once. The final performance is computed as the average accuracy across all folds.

This validation strategy mimics real-world industrial applications, where a trained model must generalize to unseen tools. By ensuring that the test set contains data from a tool not included in training, this setup evaluates the model’s ability to adapt to new cutting conditions and variations in tool wear progression.

## 4 Experimental Design, Materials and Methods

The Nonastreda dataset was collected from an industrial milling process using a shaft end milling tool operating under real manufacturing conditions. During the milling process, 3-axis cutting forces were captured at 1 kHz with a piezoelectric dynamometer (Kistler 9257B) and high-resolution images were recorded with a Keyence VHX-S15F digital microscope to model industrial tool wear progression. The machining was performed on C45 tempered steel workpieces using an end mill tool following a linear machining path. The primary health indicator monitored was flank wear.

### 4.1 Force signal acquisition system

The force measurement system consisted of multiple interconnected industrial components:

• An industrial PC (Beckhoff C6920) for decoding and storing signals.
• EtherCAT bus-coupler (Beckhoff EK1100) and amplifier for signal conditioning and transmission.
• Kistler 9257B dynamometer (factory-calibrated with linearity ≤ 0.1 % FSO and cross-talk ≤ 1.3 %) placed beneath the workpiece holder to measure cutting forces in the x, y, and z axes.

**Fig. 6:**
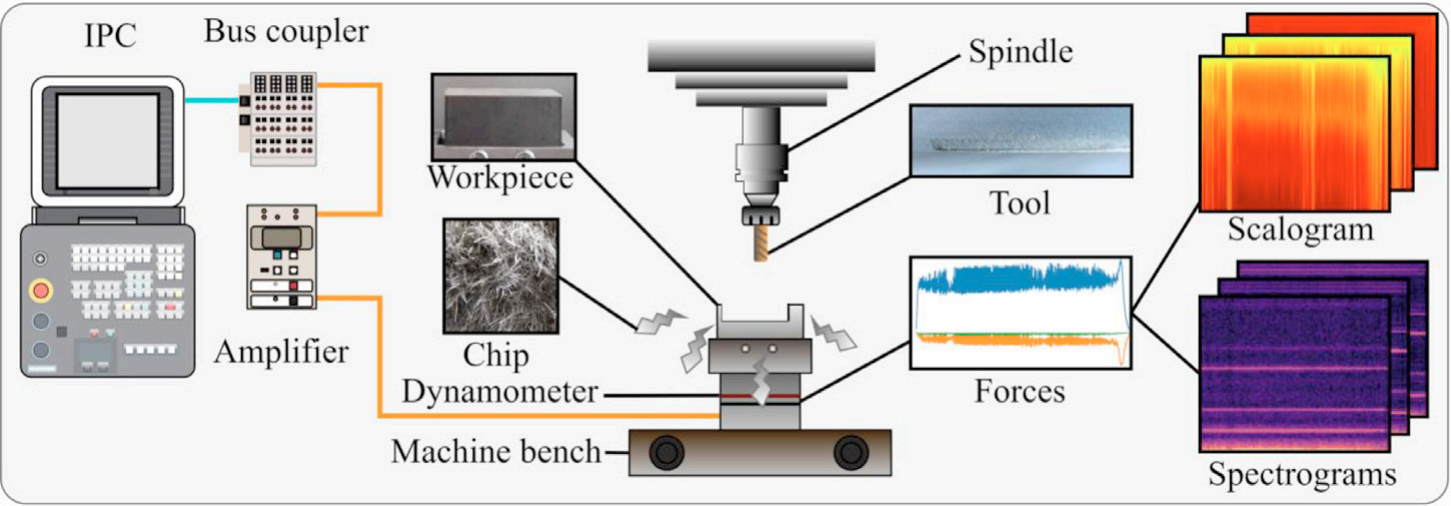
Nonastreda dataset collection setup.

The force signals were collected in real time and later transformed into scalograms and spectrograms for frequency-domain analysis. Fig. 6 illustrates the complete dataset collection setup, highlighting the sensors and recording points for each data modality.

### 4.2 Image acquisition system

The tool wear images were obtained using an industrial profile measurement microscope (Keyence VHX-S15F, expanded uncertainty of ±2 µm) positioned at a dedicated measurement station. The image acquisition process involved the following steps:

• After completing a machining pass, the tool was removed from the spindle.
• The tool was transferred to the microscope measurement stand.
• A high-resolution image of the tool’s cutting edge was captured.
• An expert operator measured the flank-wear width and mapped each measurement to one of three wear classes:
  ○ Sharp (Class 1): 0 ≤ flank-wear < 71 µm
  ○ Used (Class 2): 71 ≤ flank-wear < 110 µm
  ○ Dulled (Class 3): flank-wear ≥ 110 µm

**Fig. 7:**
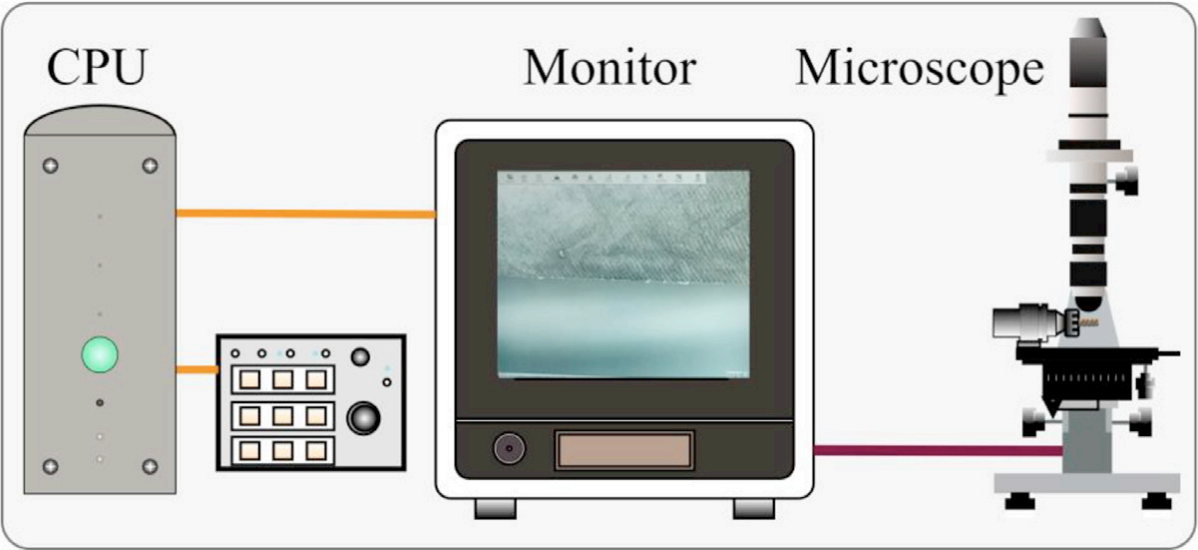
Nonastreda microscope stage setup.

These flank wear width serves as the regression labels, while the class assignments provide the corresponding classification labels. Additionally, images of the material chip and workpiece surface were collected to provide complementary visual data. Fig. 7 presents the microscope measurement setup, consisting of the CPU, monitor, and industrial microscope used for image acquisition.

## Limitations

The Nonastreda dataset consists of 512 samples from 10 tools, which may be relatively small for training deep learning models. To address this, augmentation techniques can be applied, and 10-fold cross-validation is recommended. This limitation also motivated us to develop data-efficient, lightweight multimodal deep learning models, available in our github repository.

The machining path is linear, which simplifies real-world milling operations. While this setup provides a controlled environment for initial machine learning model development, future datasets could include more complex machining paths for enhanced generalization.

Only one tool type, one workpiece material (C45 tempered steel), and a single set of machining parameters (spindle speed, feed rate) were used during data collection. This controlled setup ensures consistency but does not capture variations found in diverse industrial settings. Future expansions could incorporate multiple tools, materials, and cutting conditions to enhance dataset diversity.

For time-series force signals, a strong drift in the z-axis (Fz) was observed as presented in Fig. 4. Although this was mitigated by resetting the recording device, it resulted in zeroing the force signal after each 1–3 instances. As a result, the Fz signal primarily reflects sequential progression rather than absolute tool wear. The dataset offers flexibility for researchers to (1) exclude the Fz signal, (2) apply de-drifting techniques, or (3) utilize this pattern as implicit sequence encoding, similar to time-encoding methods used in Transformer-based architectures.

The labels were assigned manually, introducing the possibility of human error. To address this, the github repository includes original tool wear measurements (microscope images and manual annotations), allowing researchers to refine or modify labels as needed.

Additionally, the dataset does not provide segmentation labels for tool images or cutting edge line detection, which could be valuable for future research in computer vision-based tool wear analysis.

Although nine sensor modalities are included, other potentially useful signals such as machine power, current, torque, acoustic emission, or temperature sensors were not recorded. Expanding the dataset with these modalities could further enhance tool wear prediction models.

Finally, deep learning experiments using 10-fold cross-validation (available in our repository) indicate an approximate accuracy of 75 %, suggesting significant room for improvement in model architectures, feature extraction, and multimodal fusion techniques.

## Ethics Statement

Authors confirm that they have read and follow the ethical requirements for publication in Data in Brief and confirming that the current work does not involve human subjects, animal experiments, or any data collected from social media platforms

## CRediT Author Statement

**Hubert Truchan:** Writing, data curation, **Zahra Ahmadi:** Supervision

## Data Availability

Mendeley DataNonastreda: Multimodal Dataset for Identifying Tool Wear Condition (Original data).
